# Targeted therapy in advanced non-small cell lung cancer: current advances and future trends

**DOI:** 10.1186/s13045-021-01121-2

**Published:** 2021-07-08

**Authors:** Umair Majeed, Rami Manochakian, Yujie Zhao, Yanyan Lou

**Affiliations:** Division of Hematology and Medical Oncology, Mayo Clinic, 4500 San Pablo Rd, Jacksonville, FL 32224 USA

**Keywords:** Advanced NSCLC, Targeted therapy, Phase I/II clinical trials, First-in-human, Lung cancer

## Abstract

Lung cancer remains the leading cause of cancer-related mortality in both men and women in the US and worldwide. Non-small cell lung cancer is the most common variety accounting for 84% of the cases. For a subset of patients with actionable mutations, targeted therapy continues to provide durable responses. Advances in molecular and immunohistochemical techniques have made it possible to usher lung cancer into the era of personalized medicine, with the patient getting individualized treatment based on these markers. This review summarizes the recent advances in advanced NSCLC targeted therapy, focusing on first-in-human and early phase I/II clinical trials in patients with advanced disease. We have divided our discussion into different topics based on these agents' mechanisms of action. This article is aimed to be the most current review of available and upcoming targeted NSCLC treatment options. We will also summarize the currently available phase I/II clinical trial for NSCLC patients at the end of each section.

## Background

Lung cancer remains the number one cause of cancer-related death worldwide. Overall, lung cancer causes more deaths than breast, prostate, colorectal, and brain cancers combined [1]. In 2021, an estimated 235,760 new lung cancer cases will be diagnosed in the US, and 131,880 people will die from this disease [2]. Non-small cell lung cancer (NSCLC) is the most common type of lung cancer, accounting for more than two-thirds of the cases, with most patients (84%) having advance disease at the time of diagnosis [3]. Identification of targetable alteration (i.e., EGFR, ALK, PI3K/AKT/mTOR, RAS-MAPK, RET, MET, BRAF, and NTRK/ROS1) in patients with advanced NSCLC has evolved its treatment paradigm [4]. The approval and adoption of agents targeting these alterations has contributed to the decline in incidence-based mortality from 35% among men with NSCLC diagnosed in 2001 to 26% among those diagnosed in 2014. Similar patterns have been found among women with NSCLC [5]. Despite these new therapeutic options for patients with advanced NSCLC, there continues to be significant challenges as resistance development and disease progression occurs in most of these patients [6]. This has led to research in identifying drugs that can overcome these resistance pathways. Next-generation sequencing, which can be performed on the tumor tissue and circulating tumor DNA (ctDNA) in the blood, is now the standard of care for all patients with advanced NSCLC [7]. It helps in the rapid identification of actionable mutations and resistance mechanisms.

In this review after highlighting the different driver genomic alterations and their relative frequencies in advanced NSCLC we summarize the clinical efficacy and safety of FDA approved targeted therapies. We then discuss the recently published data on the first-in-human clinical trials and some of the most promising drugs in the pipeline for this disease. Literature was searched for first-in-human, phase I and phase II clinical trials in NSCLC using PubMed, Google Scholar, and the American Society of Clinical Oncology (ASCO) meeting abstracts. Each study was individually reviewed, and data points have been summarized. Finally, we present summary of ongoing clinical trials in a tabulated fashion at the end of each section.

### Predictive biomarkers in advanced NSCLC

Predictive biomarkers in NSCLC include anaplastic lymphoma kinase (ALK) fusion oncogene, ROS proto-oncogene 1 receptor tyrosine kinase (ROS1) gene fusions, sensitizing endothelial growth factor receptor (EGFR) gene mutations, BRAF V600E point mutations, neurotrophin tyrosine kinase (NTRK) gene fusions, c-mesenchymal-epithelial transition factor (c-MET) exon 14 (METex14) skipping mutations and RET rearrangements [8]. Table 1 includes the relative frequencies and most common types of these mutations in different population subgroups along with drugs of interest. Table 2 highlights select FDA approved targeted agents with corresponding clinical trials, efficacy, and common adverse effects.

**Table 1:** Distribution of actionable mutations in advanced lung adenocarcinoma and available targeted therapies [9–18]

Actionable mutation	Common Subtypes	Frequency in different populations	Targeted therapies
KRAS	G12C, G12V, G12D	Caucasian: 13–15% East Asian: 3.6% Indian: 3.9%	KRAS G12C inhibitors: Sotorasib, Adagrasib
EGFR	Deletion 19, L858R	Caucasian: 12–15% East Asian: 47–64% Indian: 22%	EGFR inhibitors: Erlotinib, Gefitinib, Afatinib, Dacomitinib, Osimertinib
ALK	EML-ALK fusion	Caucasian: 7% East Asians: 5% Indian: 3%	ALK inhibitors: Crizotinib, Ceritinib, Alectinib, Brigatinib, Lorlatinib ALK, ROS1 and pan-TRK inhibitor: Entrectinib
MET	Exon 14 skipping mutation MET amplification	Caucasian: 2.1–4.5% East Asian: 0.9–4%	MET, ALK, and ROS1 inhibitor: Crizotinib MET inhibitors: Capmatinib, Tepotinib
BRAF mutations	V600E	Caucasian: 2.6% East Asian: 1.7% Indian: 1.5–3.5%	BRAF + MEK inhibition: Dabrafenib + Trametinib
RET	RET-KIF5B	Caucasian: 1–2% East Asian: 1%	RET inhibitors: Selpercatinib, Pralsetinib
ROS1	Variable fusion partners	Caucasian:0.7–1.7% East Asian: 0.8% Indian: 2.8%	Crizotinib, Ceritinib, Lorlatinib, Entrectinib, Repotrectinib, Taletrectinib
NTRK	NTRK 1, 2, 3 with different fusion partners	Caucasian: 0.2% East Asian: 0.3% Indian: 0.7%	Pan-TRK, ALK and ROS1 inhibitor: Entrectinib Pan-TRK inhibitor: Larotrectinib
HER2	HER2 amplification HER2 Exon 20 mutation	Caucasian: 2–4% East Asian:1.3% Indian: 1.5%	Antibody drug conjugates: ado-trastuzumab emtansine, trastuzumab deruxtecan HER2 Exon 20 inhibitors: Mobocertinib, Poziotinib

KRAS: kirsten rat sarcoma viral oncogene homolog; EGFR: epidermal growth factor receptor; ALK: anaplastic lymphoma kinase; EML4: echinoderm microtubule-associated protein-like 4; BRAF: v-Raf murine sarcoma viral oncogene homolog B; HER2: human epidermal growth factor receptor 2; ROS1: c-ros oncogene 1; RET: rearranged during transfection; KIF5B: kinesin family member 5B gene; MET: c-MET; NTRK: neurotrophic tyrosine receptor kinase

**Table 2:** FDA approved targeted agents for advanced NSCLC with corresponding clinical trials, efficacy, and safety data

Actionable mutation	FDA approved therapy (citation)	Clinical trial^π^ (phase)	Comparator	ORR (%)	mPFS (months)	mOS (months)	Adverse effects
KRAS	Sotorasib	CodeBreaK 100 (I)	No	32%	6.3	12.5	Diarrhea, nausea, elevated LFT’s, fatigue
EGFR	Erlotinib	EURTAC (III)	chemotherapy	64%	9.7	22.9	Fatigue, rash, diarrhea
	Gefitinib	NEJ002 (III)	Carboplatin/Paclitaxel	74%	10.8	27.2	Rash, diarrhea
	Afatinib	LUX-Lung 3 (III)	Cis/Pemetrexed	56%	11.1	28.2	Rash, diarrhea, paronychia
	Dacomitnib	ARCHER 1050 (III)	Gefitinib	75%	14.7	34.1	Diarrhea, paronychia, rash
	Osimertinib	FLAURA (III)	Erlotinib/Gefitinib	80%	18.9	38.6	Rash, diarrhea, pneumonitis
ALK	Crizotinib	PROFILE 1014 (III)	Platinum/Pemetrexed	74%	10.9	NR	Vision disorder, diarrhea, edema
	Certinib	ASCEND-4 (III)	Platinum/Pemetrexed	73%	16.6	51.3	Diarrhea, nausea, vomiting
	Alectinib	ALEXALEX (III)	Crizotinib	83%	25.7	Immature	Elevated LFT’s, CPK elevation, anemia
	Brigatinib	ALTA 1L (III)	Crizotinib	74%	24	47.6	Elevated CPK and LFT’s
	Ensartinib^ǂ^	eXALT-3 (III)	Crizotinib	75%	25.8	Immature	Rash, pruritis, edema
	Lorlatinib	B7461006 (III)	Crizotinib	76%	NR	Immature	Hyperlipidemia, edema, increased weight
MET Exon 14 skipping mutation	Capmatinib	GEOMETRY-mono-1 (II)	No	41% (68%) *	5.4 (12.4)*	NA/NA	Peripheral edema, nausea
	Tepotinib	VISION (II)	No	46%	8.5	Immature	Peripheral edema
MET amplification	Capmatinib	GEOMETRY-mono-1 (II)	No	29% (40%)*	4.1 (4.2)*	NA/NA	Peripheral edema, nausea
BRAF mutations	Dabrafenib + Trametinib	BRF113928 (II)	No	64% (68%)*	10.8 (10.2)*	17.3 (18.2)*	Pyrexia, LFT elevation, HTN
RET	Selparcatinib	LIBRETTO-001 (II)	No	64% (85%) *	16.5 (NR)	NR/NR	Dry mouth, diarrhea, HTN
	Pralsetinib	ARROW (II)	No	61% (70%)*	16.5 (13)*	NA/NA	LFT elevation, anemia
ROS1	Crizotinib	PROFILE 1001 (I)	No	72.4%	19.3	51.4	Vision disorder, nausea, edema
	Certinib	NCT01964157(II)	No	62% (67%)*	9.3 (19.3)*	24	Diarrhea, nausea, anorexia
	Lorlatinib	NCT01970865 (I-II)	No	41% (62%)*	8.5 (21)*	NA	Dyslipidemia
	Entrectinib	STARTRK-1, STARTRK-2, ALKA-372–001 (I-II)	No	77%	19	NR	Weight gain, neutropenia
NTRK	Larotrectinib	LOXO-TRK-14001 (I-II)	No	70%	NA	NA	LFT elevation, neutropenia, anemia
	Entrectinib	ALKA, STARTRK-1, STARTRK-2 (I-II)	No	70%	NA	NA	Dysgeusia, constipation, fatigue
HER2	T-DM1^ǂ^	NCT02675829 (II)	No	44%	5	NA	Infusion reactions, thrombocytopenia
	T-DXd^ǂ^	DESTINY-Lung01 (II)	No	62%	14	NA	Neutropenia, anemia, ILD

NA: Not available, NR: Not reached

*Indicates data for treatment naïve patient

^ǂ^Drugs not yet approved by FDA

^π^Citations for each trial mentioned in text or can be accessed by clicking the trial name

#### EGFR inhibitors

EGFR mutations such as exon 19 deletions (EX19del) and exon 21 (L858R) point mutations are oncogenic drivers in around 20% of patients with lung adenocarcinoma. FDA-approved EGFR TKIs in the first-line metastatic NSCLC setting are included in Tables 1 and 2. Osimertinib is now the standard of care for untreated EGFR mutant (ex19del or L858R) advanced NSCLC due to its superior efficacy and tolerability [19].

### Resistance mechanisms

**Fig. 1:**
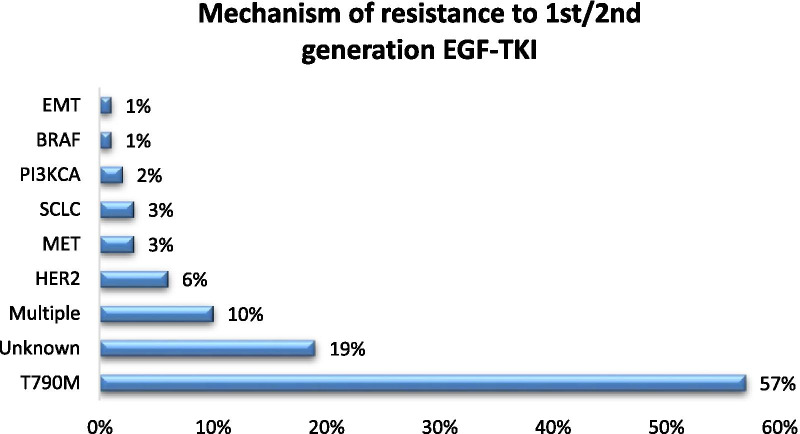
Mechanisms of acquired resistance to first-generation tyrosine kinase inhibitors (gefitinib and erlotinib) [22]. EGFR, epidermal growth factor receptor; HER2, human epidermal growth factor receptor 2; MET, mesenchymal–epithelial transition factor; EMT, epithelial–mesenchymal transition; SCLC, small-cell lung cancer

**Fig. 2:**
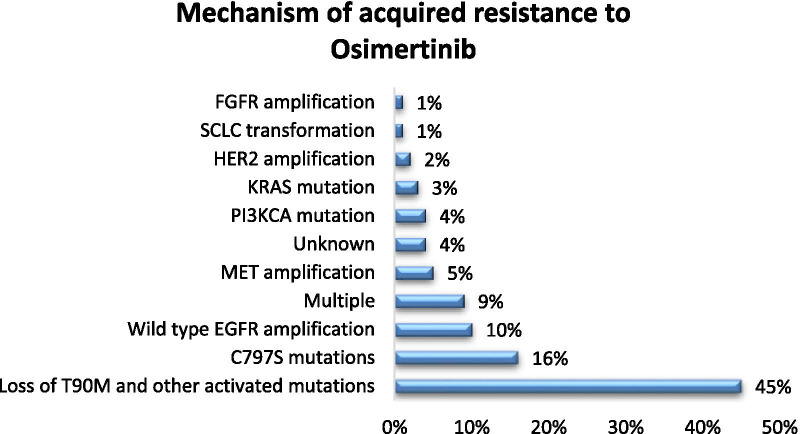
Mechanisms of acquired resistance to osimertinib [22]. EGFR, epidermal growth factor receptor; MET, mesenchymal-epithelial transition factor; HER2, human epidermal growth factor receptor 2; FGFR1, fibroblast growth factor receptor 1; KRAS, Kirsten rat sarcoma viral oncogene homolog; PIK3CA, phosphoinositide-3-kinase P110α catalytic subunit; SCLC, small-cell lung cancer

Figures 1 and 2 below summarize mechanism of acquired resistance to 1^st^/2^nd^ generation TKI and osimertinib respectively. There is currently no FDA-approved target therapy for patients who have progressed after osimertinib. The standard of care is to treat such patients with chemotherapy or chemotherapy plus immunotherapy such as Impower 150 regimen. A phase 2 ORCHARD trial (NCT03944772) examines the optimal treatment for patients with EGFR mutated NSCLC depending on their underlying resistance mechanism to frontline osimertinib. Checkmate 722 (NCT02864251) is a phase III trial of nivolumab with chemotherapy or ipilimumab vs chemotherapy in EGFR-mutant, T790M-negative stage IV or recurrent NSCLC after progression on EGFR TKI therapy [20]. Similarly KEYNOTE-789 (NCT03515837) is a phase III study looking at pemetrexed-platinum with or without pembrolizumab in EGFR mutated NSCLC with resistance to TKI therapy [21].

### Third generation EGFR inhibitors in development

Lazertinib (YH25448) is a highly mutant selective TKI that targets EGFR mutations, including T790M. In a Phase I/II study, it was found to have an overall response rate (ORR) of 57.9% with a disease control rate of 89.5% in patients who had progression of disease on first or second-generation EGFR TKI with a defined tumor T790M mutation status. Median progression-free survival (PFS) was 11.0 months, and the median duration of response (DOR) was 15·2 months. Treatment-emergent adverse effects (TEAEs) included rash, pruritus, and paresthesia [23].

Olmutinib was studied in an open label, international phase 2 study, in patients with EGFR mutated NSCLC who failed ≥ 1EGFR TKIs with confirmed T790M mutation. 162 patients were enrolled from 68 sites in 9 countries. The median treatment duration was 6.5 months. Overall, 46.3% of patients had a confirmed objective response (all partial responses). The confirmed disease control rate (DCR) for all patients was 86.4%. The median duration of objective response (DOR) was 12.7 months. Estimated median progression-free survival (PFS) was 9.4 months, and estimated median overall survival was 19.7 months [24].

Nazartinib (EGF816) inhibits T790M or activating mutations or both. It was studied in a phase I trial to determine the safety in 180 patients with varying EGFR mutation status and previous therapy. The recommended phase II dose was found to be 150 mg once daily. The most common AEs were diarrhea and rash [25]. In a phase II study of treatment naïve patients with EGFR-mutant NSCLC, 45 patients received 150 mg daily of nazartinib. Overall response rate by blinded independent review committee (BIRC) was 69% with a median PFS of 18 months. Median overall survival (OS) was not evaluable and at 33 months, 56% of pts were alive. Most frequent adverse effects (AEs) were diarrhea (47%), maculopapular rash (38%) and pyrexia (29%). Most frequent grade 3/4 AEs were maculopapular rash and increased lipase [26]. Nazartinib is also being studied in combination with gefitinib (NCT03292133) and trametinib (NCT03516214).

Aflutinib (AST2818) has been studied in a phase IIB single-arm study in patients with EGFR T790M mutated NSCLC after progression on first/second-generation EGFR-TKIs therapy or primary EGFR T790M mutation. In 220 enrolled patients, ORR was 73.6% (95% CI 67.3–79.3) with a median PFS of 7.6 months. The most common AEs were, cough (15%), upper respiratory infection (15%) and AST elevation (15%). Grade 3–5 AEs occurred in 42 (19.1%) patients, the most common one was elevated γ-glutamyltransferase [27]. A phase II trial with aflutinib is currently enrolling (NCT03502850).

### Next generation of EGFR inhibitors

The 4th generation of EGFR TKIs, including EAI045 and BLU-945, are currently being studied to overcome C797S which is the most significant on-target resistance mechanism to osimertinib [28]. EAI045 is the first allosteric inhibitor that targets T790M and C797S EGFR mutations. It has shown efficacy in combination with cetuximab in mouse models [29]. BLU-945 is another fourth-generation EGFR TKI that potently inhibits triple-mutant EGFR that harbors either activated L858R or exon 19 deletion mutations, plus acquired T790M and C797S mutations [28]. A Phase I/II, open-label, first-in-human (FIH) study NCT04862780 is recruiting to evaluate the safety, tolerability, pharmacokinetics (PK), pharmacodynamics (PD), and anticancer activity of BLU-945 in EGFR-mutated NSCLC who have previously received at least 1 prior EGFR-targeted TKI including those with C797S mutation.

BDTX-189 is an irreversible small-molecule inhibitor that targets oncogenic driver mutations of EGFR, HER2, and ERBB kinases [30]. Safety and preliminary efficacy from first-in-human phase I/II trial Mastery key-01 (NCT04209465) in patients with advanced solid cancers including EGFR mutant NSCLC was reported [31]. The maximum tolerated dose (MTD) for QD (fasting) was 800 mg, with 2/6 pts with DLTs at 1200 mg due diarrhea and nausea/vomiting. The most frequent (≥ 20%) related adverse events were diarrhea (36%, 8% G3), nausea (28%, 0% G3), and vomiting (25%, 3% G3). The rate of skin disorders was 11% with the highest severity of G2 in 1 pt.

CLN-081 (TAS6417) is a potent pan-mutation-selective EGFR inhibitor with a broad therapeutic window. Pre-clinical studies have shown TAS6417 as a potent inhibitor against EGFR exon 19 deletions, L858R, T790M, G719X, L861Q, S768I, and exon 20 insertion mutations [32]. It is currently being studied in a phase I/II trial in patients with EGFR exon 20 mutation (NCT04036682). Interim results for safety and efficacy were reported in 37 heavily pre-treated patients. The most common TRAEs were rash (49%), diarrhea (24%), paronychia (16%), nausea (14%), stomatitis (14%), and dry skin (11%). Grade 3 TRAEs included anemia (5%), diarrhea (3%), and increased alkaline phosphatase (ALP) (3%). Among the 25 response evaluable pts, 10 (40%) had a partial response (PR) and 14 (56%) had stable disease [33].

DZD9008 is a potential EGFR TKI for NSCLC patients with EGFR or HER2 Exon20 insertion (exon20ins) and other activating mutations [34]. Two ongoing phase I/II clinical trials (NCT03974022 and CTR20192097) are currently enrolling patients. Preliminary safety and efficacy results from these phase I studies of DZD9008 were presented in ASCO 2021[35]. Ninety seven NSCLC patients with EGFR or HER2 mutations were dosed (range 50 mg to 400 mg, once daily) with DZD9008, 59 of these had EGFR exon20ins. DZD9008 was well tolerated up to 400 mg (MTD) once daily. The DLTs were diarrhea and cardiac arrhythmia. The most common TEAEs were diarrhea (grade 3, 5.2%) and skin rash (grade 3, 1%). In a heavily pretreated population, at the RP2D dose of 300 mg once daily, the objective response rate was 48.4% (15/31), and disease control rate (DCR) was 90.3% (28/31) [35].

Tarloxotinib is a prodrug of a pan-ERBB kinase inhibitor that releases a potent metabolite (Tarloxotinib-E) in hypoxic conditions. It preferentially delivers the active moiety to the tumor over normal tissues. The first analysis of RAIN-701 trial included patients with advanced NSCLC with an EGFR Exon 20 insertion (Cohort A) or HER2 activating mutation (Cohort B) with progression after platinum-containing chemotherapy. The disease control rate for all evaluable patients was 60% (12/20). The grade 3 TEAEs included prolonged QTc (34.8%), rash (4.3%), diarrhea (4.3%), and increased ALT (4.3%) [36]. Patients are currently being enrolled in NCT03805841.

Amivantamab (JNJ-372) is a novel, fully human anti-EGFR-MET bispecific antibody which targets both EGFR and MET driven disease [37]. Preliminary results of pts with advanced NSCLC harboring exon 20 insertion (exon20ins) mutations in the ongoing CHRYSALIS study led to its accelerated approval by FDA for this subset of patients in May 2021 [38]. 50 patients with exon20ins received amivantamab. 39/50 patients were response evaluable with a median follow up of 4 months. In the response evaluable patients ORR was 36%, clinical benefit rate was 67%, mPFS was 8.3 months and duration of response was 10 months. The most common adverse events were rash (72%), infusion related reactions (60%), and paronychia (34%). Grade ≥ 3 were reported in 36% of the patients [37].

Poziotinib is a potent and irreversible TKI with a structure that can overcome the steric hindrance of the exon 20 limited binding pocket. In the ZENITH20 trial Poziotinib showed excellent CNS activity in patients with EGFR or HER2 exon 20 mutations with ORR of 22.2% (8/36), DCR of 88.9% (32/36) and intra cranial complete response in 3/36 patients [39]. A phase II trial NCT03318939 is currently recruiting advanced NSCLC with EGFR or HER2 Exon 20 Insertion Mutation.

Mobocertinib (TAK-788) is a potent oral TKI targeting EGFR ex20ins mutations and has breakthrough therapy designation in the US for post platinum based chemotherapy pts with EGFR ex20ins positive mutant NSCLC [40]. This 3-part, open-label, multicenter study (NCT02716116) has dose-escalation/expansion and extension (EXCLAIM) cohorts. Efficacy and safety data for 114 platinum-pretreated pts (PPP) and 96 pts from EXCLAIM safety cohort was presented in ASCO 2021. Among PPP pts confirmed ORR was 28%, including 1 CR. In this group the DCR was 78%, median DOR was 17.5 mo. In the EXCLAIM cohort (*n* = 96) 49% had received ≥ 2 prior lines. Confirmed ORR was 25%, with 1 CR; DCR was 76% and median DOR was not reached. Most common TRAEs were diarrhea (91%), rash (45%) and paronychia (38%). AEs leading to discontinuation in > 2% were diarrhea (4%) and nausea (4%) [40].

### EGFR therapy in combination with other targeted agents

Amivantamab is also being evaluated in NCT02609776 clinical trial as a monotherapy or combination with Lazertinib or chemotherapy (Carboplatin/Pemetrexed) in EGFR or MET mutant/amplified NSCLC. The preliminary responses were achieved in the third generation EGFR TKI-relapsed disease, including C797S, cMet amplification, and Exon20ins disease [41]. Updated results of amivantamab and lazertinib combination in osimertinib relapsed patients with EGFR mutant NSCLC were presented in ASCO 2021. Of the 45 osimertinib-relapsed patients 36% had a confirmed response (1 complete response and 15 partial responses). At a median follow up of 8.2 months 20 out of 45 patients (44%) remain on treatment. The mPFS was 4.9 months. An IHC-based approach identified pts most likely to benefit from the combination regimen with 9/10 (90%) IHC high (combined EGFR + MET H score > 400) pts and only 1/10 IHC low pts responding to treatment [42].

Osimertinib and savolitinib (MET TKI) combination was studied in a phase 1b study with advanced, MET-amplified, EGFR mutant NSCLC who had progressed on EGFR TKIs. It was shown to have an acceptable risk–benefit profile and encouraging anti-tumor activity in MET-amplified, EGFR mutant positive, advanced NSCLC patients [43]. Combinations using the third-generation TKI such as osimertinib and an earlier generation TKI dacomitinib is being studied (NCT03810807) in the hope to prevent different acquired EGFR alterations. In the INSIGHT 2 study, combination of osimertinib and tepotinib is being studied in patients with EGFR mutant NSCLC with acquired resistance to first line osimertinib due to MET amplification (NCT03940703) [44].

The promising synergy between EGFR and VEGFR inhibitors was seen in phase 3 RELAY study (NCT02411448), in which the addition of ramucirumab to erlotinib lead to a median PFS of 19.4 months (95% CI 15.4–21.6) compared with 12.4 months (95% CI 11.0–13.5) for erlotinib alone without any new adverse events [45]. Based on this data, the FDA approved this combination in May 2020 [46]. In a phase 2 randomized clinical trial of 81 patients with NSCLC with EGFR T790M mutation, osimertinib plus bevacizumab failed to show prolongation of progression-free survival and overall survival compared with osimertinib alone [47]. Currently there is another ongoing trial looking at the combination of osimertinib with ramucirumab (phase II; NCT03909334) [48]. Interim results from a median follow-up time of 25 months show an ORR of 76%. Median DoR was 13.4 months (90% CI 9.6–21.2 months). Median PFS was 11.0 months (90% CI 5.5–19.3 months). Common Grade 3 or higher TRAEs were hypertension (8%) and platelet count decreased (16%) [49]. Table 3 summarizes current ongoing phase I/II trials with EGFR inhibitors.

**Table 3:** Current ongoing early phase I/II trials with EGFR inhibitors

EGFR inhibitors				
Drug name	Mechanism of action	Clinical trial (phase)	Study design	Disease
Aflutinib (ASK120067)	EGFR T790M Inhibitor	NCT03502850 (1/2)	Monotherapy	EGFR mutant NSCLC progression on first line therapy due to T790M
BDTX-189	EGFR/HER2 Inhibitor	NCT04209465 (1/2)	Monotherapy	EGFR and HER2/3 mutated solid cancers
CLN-081 (TAS6417)	Pan-EGFR Inhibitor	NCT04036682 (1/2)	Monotherapy	EGFR exon 20 mutated NSCLC
D-0316	EGFR T790M Inhibitor	NCT04206072 (2/3)	Monotherapy	EX19del, L858R mutated NSCLC adenocarcinoma
Dacomitinib	Pan-EGFR inhibitor	NCT03755102 (1)	Monotherapy or in combination with Osimertinib	EGFR mutant NSCLC
DZD9008	Wild type EGFR and HER2 Inhibitor	NCT03974022 (1/2)	Monotherapy	EGFR/HER2 mutated NSCLC
FCN-411	EGFR, HER2 and HER4 Inhibitor	NCT03420079 (1/2)	Monotherapy	EGFR mutant NSCLC
JNJ-61186372	Bispecific antibody binding to EGFR and cMET	NCT02609776 (1)	Monotherapy or in combination with Lazertinib and Carboplatin/Pemetrexed	EGFR or MET mutant/amplified NSCLC
Nazartinib (EGF816)	Irreversible selective mutant specific EGFR Inhibitor	NCT03292133 (2)	Combination with Gefitinib	EGFR mutant NSCLC
Nazartinib (EGF816)	Irreversible selective mutant specific EGFR Inhibitor	NCT03516214 (1)	Combination with Trametinib	EGFR mutant (del19 or p.L858R) NSCLC
Necitumumab	Anti EGFR mAB	NCT02496663 (1)	Combination with Osimertinib	EGFR mutant NSCLC
Osimertinib	Potent mutant EGFR Inhibitor	NCT03434418 (2)	Monotherapy	NSCLC with uncommon EGFR mutations (EGFR 18;G719X, EGFR 20;S7681, EGFR 21;L861Q)
Tarloxotinib	Hypoxia activated prodrug (TH-4000) wild type EGFR Inhibitor	NCT03805841 (2)	Monotherapy	EGFR exon 20 and HER2 activating mutant NSCLC
WSD0922-FU	EGFR/EGFRvIII Inhibitor	NCT04197934 (1)	Monotherapy	EGFR mutant NSCLC
ZN-e4 (KP-673)	EGFR Inhibitor including T790M	NCT03446417 (1/2)	Monotherapy	EGFR mutant NSCLC

#### ALK Inhibitors

Rearrangements in the anaplastic lymphoma kinase (ALK) are found in 3–7% of the patients in NSCLC patients [50]. Crizotinib was the first FDA-approved ALK inhibitor. Since then ceritinib, alectinib, brigatinib, and lorlatinib have been approved as detailed above in Tables 1 and 2.

First-line brigatinib evaluated against crizotinib in advanced ALK + NSCLC in the ALTA-1L trial was proven to be superior to crizotinib by BIRC-assessed PFS (24.0 v 11.0 months). Brigatinib was better tolerated with a delayed median time to worsening of global health status/QoL scores compared with crizotinib without any new safety concerns [51].

First-line lorlatinib was evaluated against crizotinib in a phase 3 randomized CROWN trial. ORR was 76% in the lorlatinib group vs 58% in the crizotinib group. The percentage of patients without cancer progression at 12 months was 78% in lorlatinib group as compared to 39% in crizotinib group. More grade 3 or 4 adverse events (primarily altered lipid levels) were found in lorlatinib group than crizotinib group (72% vs. 56%) [52].

SAF-189 s is a novel selective ALK inhibitor with CNS penetration. In a phase I/II study, 36 patients with advanced ALK-positive NSCLC, 22 had CNS metastatic disease, and 26 patients had progressed on prior TKIs therapy. SAF-189 s was orally administered under fasting condition at doses ranging from 20–210 mg once daily in a 21-day cycle. All 34 in efficacy analysis set achieved tumor shrinkage, with 17 confirmed PR (50%) and 4 unconfirmed PR (11.7%) [53]. The most common drug-related events were nausea (38.9%), vomiting (27.8%) and QT prolongation (25.0%). Only one DLT of grade 3 blood glucose increase occurred at 210 mg [53].

TQ-B3101 inhibits ALK, ROS1, and MET. In a phase I study, 27 lung cancer patients (19 ALK + , 6 ROS1 + , 2 MET amplified) patients were enrolled. ORR was 87.5% in the 350 mg bid cohort (7/8). In 8 patients with brain metastasis, ORR was 62.5% (5/8). The most common grade 3 adverse effects were neutropenia (20%) and ALT increase (6.67%) [54]. Currently, TQ-B3101 is studied in a phase 2 trial in subjects with ALK-positive NSCLC that have progressed on crizotinib (NCT04056572).

TPX-0131 is a next generation ALK inhibitor that can bind completely within the ATP binding boundary to overcome a variety of ALK resistant mutations, especially SFM G1202R and compound mutations L1196M/G1202R. TPX-0131 is more than 100-fold more potent against G1202R than lorlatinib in cell proliferation assays [55]. It is currently being evaluated in a phase I/II trial (NCT04849273) in patients with ALK + advanced or metastatic NSCLC.

Ensartinib (X-396) is a highly selective and potent ALK inhibitor. The interim analysis of phase III eXalt3 study (NCT02767804) comparing ensartinib to crizotinib was recently presented. Median PFS was 25.8 months with ensartinib vs 12.7 months with crizotinib [56].

Safety and efficacy of crizotinib was studied in combination with bevacizumab in ALK/ROS-1/c-MET positive NSCLC in an open label, single arm, prospective study. The median PFS and DOR of the patients with ALK rearrangement were 13.9 and 14.8 months respectively. The most two common treatment-related adverse events were fatigue (28.6%) and rash (21.4%) [57].

Brigatinib in combination with bevacizumab (NCT04227028) and alectinib in combination with cobimetinib (NCT03202940) are currently under investigation.

The National Cancer Institute's ALK Master Protocol (NCT03737994) will prospectively match patients to appropriate ALK TKIs based on the underlying ALK-resistance mutation. Table 4 summarizes the ongoing phase I/II clinical trials involving ALK inhibitors in patients with advanced NSCLC.

**Table 4:** Current ongoing early phase I/II trials with ALK inhibitors in advanced NSCLC

ALK inhibitors				
Drug name	Mechanism of action	Clinical trial (Phase)	Study design	Disease
Alectinib	ALK inhibitor	NCT03202940 (1/2)	Combination with Cobimetinib	Advanced ALK rearranged NSCLC with progression on Alectinib
Brigatinib	Dual ALK and EGFR inhibitor	NCT02706626 (2)	Monotherapy	Advanced ALK rearranged NSCLC with progression on ALK inhibitors
Brigatinib	Dual ALK and EGFR inhibitor	NCT04227028 (1)	Combination with Bevacizumab	Advanced ALK rearranged NSCLC with progression on ALK inhibitors
Ensartinib	Selective ALK inhibitor	NCT04415320 (2)	Monotherapy	Advanced ALK rearranged NSCLC
TPX-0131	ALK inhibitor	NCT04849273 (1/2)	Monotherapy	Advanced ALK rearranged NSCLC with progression on at least one prior 2^nd^ or 3^rd^ gen ALK TKI
TQ-B3139	Multi-target inhibitor of MET/ALK/ROS	NCT04056572 (2)	Monotherapy	Advanced ALK rearranged NSCLC with progressive disease on Crizotinib

#### MET inhibitors

C-MET is a hepatocyte growth factor (HGF) receptor that is involved in cell survival and proliferation. Oncogenic mutations in MET include MET exon 14 skipping mutations (3–4% of NSCLC adenocarcinomas), MET gene copy number (GCN) gain or amplification, and MET protein overexpression. These patients generally have poor responses to immunotherapy, even if the expression of PD-L1 is high [58]. There are currently two FDA-approved MET inhibitors that can be used as first-line therapy in metastatic NSCLC with MET exon 14 skipping mutations detailed above in Table 2.

FDA has approved capmatinib as the first-line in patients with MET exon 14 skipping mutations based on the GEOMETRY mono-1 trial [59]. Capmatinib has also shown activity in patients with high-level MET amplification (GCN ≥ 10) in treatment naïve patients [60]. Updated results from the GEOMETRY mono-1 study in treatment naïve MET exon 14-mutated advanced NSCLC (cohort 7 and cohort 5b) were presented in ASCO 2021 [61]. The median PFS was 10.8 months and median OS was 20.8 months. 98.4% of pts reported AEs (68.6% Grade 3/4) regardless of causality and 16.1% reported AEs leading to discontinuation. The most common AEs were peripheral edema (54.2%), nausea (45.0%), vomiting (28.2%) and increased blood creatinine (26.5%) [61].

Tepotinib, a highly selective MET inhibitor, is being evaluated in a phase II trial NCT02864992. In VISION trial, a total of 152 patients had received tepotinib, with 99 patients being followed for at least 9 months. ORR was 46% (95% confidence interval [CI], 36 to 57), with a median duration of response of 11.1 months (95% CI 7.2-could not be estimated). Tepotinib also demonstrated robust activity in pts with METex14 skipping NSCLC with metastatic brain disease [62]. Adverse events of grade 3 were reported in 28% of the patients, including peripheral edema in 7% [63]. FDA granted accelerated approval to tepotinib in February 2021 based on the above data. Tepotinib was also evaluated in patients with advanced NSCLC with MET amplification by liquid biopsy, MET gene copy number ≥ 2.5. Among 24 enrolled patients the ORR was 42% with no new safety signals [64].

Sym015, a mixture of 2 humanized antibodies, triggers MET degradation. In a sym015-01 phase I/II trial, safety and efficacy were studied in patients with MET exon 14 deletion (*n* = 12) or MET amplification (*n* = 8). Of 20 NSCLC pts, 5 had confirmed PR, and 11 had SD (DCR 80%; 6/8 METAmp and 5/12 METEx14Δ). In the safety arm with 45 patients, grade 3 or above treatment-related adverse events were reported in 13.3% pts [65].

Glumetinib is a selective MET inhibitor that was well tolerated in advanced NSCLC with MET alterations at doses up to 400 mg once daily. A phase I trial continues to enroll patients (NCT03466268).

APL-101 is a highly selective small-molecule c-Met inhibitor that targets the c-Met-dysregulated pathway in several tumors [66]. A phase I/II, multicenter, global trial, recruits patients with c-MET exon 14 skipping mutations and c-met dysregulation (NCT03175224).

REGN5093 is a human bispecific antibody that binds to two distinct epitopes of MET, blocking HGF binding and inducing MET's internalization and degradation [67]. A Phase I/II, first-in-human, multicenter study investigates the safety, tolerability, pharmacokinetics (PK), and efficacy of REGN5093 in patients with MET-altered advanced NSCLC who have received all available approved therapies (NCT04077099). Table 5 summarizes the ongoing phase I/II clinical trials involving MET inhibitors in patients with advanced NSCLC.

**Table 5:** Current ongoing early phase I/II trials with MET inhibitors

MET Inhibitor				
Drug name	Mechanism of action	Clinical trial (phase)	Study design	Disease
Bozitnib (APL-101)	c-MET receptor (HGFR) Inhibitor	NCT03175224 (1/2)	Monotherapy	c-MET altered solid tumors including NSCLC
Capmatinib (INC280)	MET Inhibition through HGFR binding	NCT04139317 (2)	Combination with Pembrolizumab	EGFR wild type and ALK negative NSCLC with high PDL1 expression
Capmatinib (INC280)	MET Inhibition through HGFR binding	NCT02414139 (2)	Monotherapy	EGFR wild type and ALK negative NSCLC with cMET alteration
Glumetinib	Small molecule MET kinase Inhibitor	NCT04338243 (1/2)	Monotherapy	T790M Mutation negative and Met amplified NSCLC
REGN5093	Bispecific antibody that Inhibits MET	NCT04077099 (1/2)	Monotherapy	MET altered NSCLC
Tepotinib	Selective MET inhibitor	NCT03940703 (2)	Combination with Osimertinib	MET amplified NSCLC with EGFR mutation
TFX-0022	MET, CSF1R, SRC kinase Inhibitor	NCT03993873 (1)	Monotherapy	NSCLC with MET alterations

#### RET inhibitors

RET gene rearrangements are found in 1–2% of NSCLC adenocarcinomas and are mutually exclusive with EGFR, ALK, or RAS mutations [68]. NSCLC with RET fusion is associated with a high risk of brain metastasis [69]. Cabozantinib and vandetanib were previously used with modest benefit and significant toxicity. However, selpercatinib and pralsetinib have recently been approved by the FDA after promising phase II clinical trial results (Table 1).

Selpercatinib (LOXO-292) is a highly selective small-molecule inhibitor of RET kinase approved by the FDA to treat NSCLC with RET fusion. In a phase II trial of 105 patients pretreated with platinum-based chemotherapy, the ORR was 64% (95% confidence interval [CI], 54% to 73%) with a median duration of response of 17.5 months. Among the 39 previously untreated patients, ORR was 85%, and 90% of the responses were ongoing at 6 months. The intracranial response was 91% (95% CI 59% to 100%). The most common adverse events were hypertension (14%) and an increase in LFTs (12%) [70]. Updated overall efficacy and safety data continues to show that selpercatinib has durable antitumor activity in pts with RET-fusion + NSCLC [71]. A global, randomized, phase 3 trial NCT04194944 (LIBRETTO-431) evaluating selpercatinib compared with standard frontline therapy is ongoing.

Pralsetinib was approved by FDA based on the phase I/II ARROW trial, which showed efficacy in patients with RET fusion–positive NSCLC with or without prior therapy [72]. Updated results from the ARROW trial show an ORR of 62% with a DCR of 91% in patients with prior platinum therapy (*n* = 126) and 79% (*n* = 68) with DCR of 93% in treatment naïve patients. The median PFS is 16.5 months in prior platinum group while it has still not matured in the treatment naïve group [73]. The most common TRAEs were increased aspartate aminotransferase (39%), anemia (35%), increased alanine aminotransferase (27%), constipation (26%) and hypertension (25%). Overall, 6% of patients discontinued treatment due to TRAEs [73]. A phase III clinical trial is currently enrolling patients (NCT04222972).

TPX-0046 is a next-generation RET inhibitor active in drug-resistant cancer models, including solvent front mutations (SFMs) mediated resistance [74]. A phase I/II clinical trial for RET inhibitor-resistant and naïve RET-driven cancers is ongoing (NCT04161391).

BOS172738 is an oral, highly potent and selective RET inhibitor [75]. It has > 300-fold selectivity against vascular endothelial growth factor receptor 2 [76]. In a phase 1 clinical trial NCT03780517 it is being studied in RET-altered advanced solid tumors including advanced NSCLC. According to the results reported BOS172738 exhibited a favorable safety profile in the studied 67 patients. The most common TEAEs were creatinine phosphokinase (CPK) increase (54%), dyspnea (34%), facial edema, aspartate aminotransferase elevation, anemia (25% each) and neutropenia, diarrhea (22% each). BOS172738 demonstrated broad anti-tumor activity with an investigator-assessed ORR of 33% (*n* = 18/54) and a NSCLC ORR of 33% (*n* = 10/30) [76]. Table 6 summarizes the ongoing phase I/II clinical trials involving RET inhibitors in patients with advanced NSCLC.

**Table 6:** Current ongoing early phase I/II trials with RET inhibitors

RET Inhibitors				
Drug name	Mechanism of action	Clinical trial (phase)	Study design	Disease
Pralsetinib (BLU-667)	RET inhibitor	NCT03037385 (1/2)	Monotherapy	RET altered solid tumors including NSCLC
Selpercatinib (Loxo-292)	RET, VEGFR1, VEGFR3 Inhibitor	NCT03157128 (1/2)	Monotherapy	RET altered solid tumors including NSCLC
TPX-0046	RET/SRC Inhibitor	NCT04161391 (1/2)	Monotherapy	RET altered solid tumors including NSCLC
BOS172738	RET inhibitor	NCT03780517 (1)	Monotherapy	RET altered solid tumors including NSCLC

#### BRAF/MEK inhibitors

BRAF is a serine/threonine kinase involving the canonical MAP/ERK signaling pathway. Dabrafenib inhibits BRAF V600E mutations, and trametinib inhibits MEK [77]. Combination of dabrafenib and trametinib is now the preferred, and the only FDA approved first-line therapy based on a phase II trial that assessed this combination in 36 patients with newly diagnosed, metastatic NSCLC and BRAF V600E mutation [78].

LXH254 is a BRAF and CRAF inhibitor with activity in MAPK-driven tumor models. Oral LXH254 was well tolerated, with antitumor activity observed in a phase I trial of 75 patients with advanced pretreated solid tumors with MAPK pathway alterations [79]. It is currently being studied in a phase IB study in combination with LTT462 (ERK1/2 inhibitor), trametinib (MEK inhibitor), and ribociclib (CDK4/6 inhibitor) in patients with advanced KRAS or BRAF Mutant NSCLC (NCT02974725).

ABM-1310 is an investigational, oral, small-molecule BRAF inhibitor undergoing study in phase I, first-in-human, open-label study in patients with advanced solid cancer, including NSCLC with BRAF V600E mutation (NCT04190628). Table 7 summarizes the ongoing phase I/II clinical trials involving BRAF/MEK inhibitors in patients with advanced NSCLC.

**Table 7:** Current ongoing early phase I/II trials with BRAF/MEK inhibitors

BRAF/MEK inhibitors				
Drug name	Mechanism of action	Clinical trial (phase)	Study design	Disease
ABM-1310	BRAF inhibitor (BRAF V600E)	NCT04190628 (1)	Monotherapy	Advanced BRAFV600 E mutated solid tumor including NSCLC
LXH254	BRAF and CRAF inhibitor	NCT02974725 (1)	Combination with LTT462, Trametinib, Ribociclib	Advanced BRAF or KRAS mutant NSCLC
Trametinib	MEK inhibitor	NCT03225664 (1/2)	Combination with Pembrolizumab	Advanced NSCLC with EGFR or ALK mutation with progression on first line therapy

#### ROS1 inhibitors

ROS1 is a distinct receptor tyrosine kinase like ALK. It is estimated to be present in 1–2% of patients with NSCLC (80). Crizotinib, entrectinib, and certinib are recommended first-line therapy options for these patients, according to the NCCN.

Entrectinib is an oral TKI that inhibits both ROS1 and TRK. Pooled data from 3 trials in 53 patients with-ROS1 positive metastatic NSCLC receiving entrectinib in the first-line showed an ORR of 77% (41/53; 95% CI 64–88%). Intracranial ORR was 55% (95% CI 32–77%). In the larger ROS1 population of 134 patients, Grade 3 and 4 adverse effects were seen in 34% of patients (81).

Lorlatinib is a third-generation TKI that targets ALK and ROS1. In a phase I-II trial with 69 patients with advanced ROS1 positive NSCLC after an estimated median duration of follow up of 21.1 months, 13 of 21 (62%; 95% CI 38–82%) TKI naïve patients and 14 of 40 (35%; 21–52%) patients previously treated with crizotinib had an objective response. The intracranial responses were achieved in 7 of 11 TKI naïve patients and 12 of 24 previous crizotinib treated patients. The most common adverse effects were hypertriglyceridemia (19%) and hypercholesterolemia (14%). No responses were seen in patients with G2032R mutations [82].

Repotrectinib is a next-generation ROS1/TRK inhibitor with > 90-fold potency against ROS1 than crizotinib. Preclinical studies have shown inhibitory activity against ROS1 resistance mutations, including SFM G2032R. In a phase 1 study, repotrectinib was well tolerated and showed a 91% overall response in TKI naïve ROS1 + NSCLC patients. 100% intracranial-ORR was observed in TKI naïve, and 75% was observed in patients with one prior TKI [83]. A global phase 2 expansion study is actively enrolling (TRIDENT-1) (NCT03093116).

Taletrectinib (AB-10) is a potent and selective ROS/NTRK inhibitor. In two phase I trials, NSCLC patients with ROS1 fusion who received taletrectinib as first line ROS1 TKI had an objective response rate (ORR) of 66.7% (6/9) and median progression-free survival (PFS) of 29.1 months [84]. Data from an ongoing phase II TRUST study (NCT04395677) of taletrectinib in Chinese NSCLC with ROS 1 fusion was presented. 22 pts had received taletrectinib treatment. Most pts (54.5%, 12/22) had prior systematic chemotherapy; 31.8% (7/22) of pts had prior crizotinib treatment. ORR by investigator among the crizotinib naïve pts with tumor assessment (*N* = 11) was 100%; 81.8% (18/22) of pts had TEAEs. 13.6% (3/22) were grade ≥ 3, including fatigue, white blood cell decrease and transaminase elevation [85]. Table 8 summarizes the ongoing phase I/II clinical trials involving ROS1 inhibitors in patients with advanced NSCLC.

**Table 8:** Current ongoing early phase I/II trials with ROS1 inhibitors

ROS1 inhibitors				
Drug name	Mechanism of action	Clinical trial (phase)	Study design	Disease
Ceritinib	ALK and ROS1 inhibitor	NCT03399487 (2)	Monotherapy	ROS 1 rearranged NSCLC
Crizotinib	ALK, HGFR, C-Met and RON Inhibitor	NCT04084717 (2)	Monotherapy	NSCLC with ROS1 rearrangement or MET activating mutation/amplification
Repotrectinib	ROS1/TRK inhibitor	NCT03093116 (2)	Monotherapy	ROS1/TRK altered NSCLC
Lorlatinib	ALK and ROS1 inhibitor	NCT02927340 (2)	Monotherapy	ALK/ROS1 rearranged NSCLC with CNS disease
Taletrectinib	ROS 1 and TRK fusion inhibitor	NCT04395677 (2)	Monotherapy	Advanced NSCLC with ROS1 fusion gene

#### NTRK gene fusion inhibitors

TRK gene fusions act as oncogenic drivers for solid tumors, including lung cancer. NTRK fusions are oncogenic driver mutations in 0.1% of patients with NSCLC [86]. Larotrectinib and entrectinib are FDA-approved therapies in the first or subsequent line for patients with NTRK gene fusions.

Larotrectinib is a TRK fusion inhibitor. In a pooled analysis, 75% of the patients with NSCLC (9/12) patients showed ORR [87]. A phase II basket trial for solid tumors with NTRK fusion, including NSCLC, is enrolling patients (NCT02576431).

Entrectinib has also been assessed in patients with NTRK gene fusion-positive NSCLC in phase 1 and 2 trials. Pooled data from three trials with ten patients showed an ORR of 70% (95% CI 35–93%; 7/10), with most patients (70%) receiving one or more prior lines of therapy [88]. A phase 2 study of patients with solid tumors, including NSCLC with NTRK and ROS1 gene alteration, is currently enrolling patients (NCT02568267).

A phase II expansion study (TRIDENT-1) testing repotrectinib is also enrolling patients with TRK gene rearrangement (NCT03093116).

NTRK1 gene encodes tropomysin receptor kinase A (TrKA) protein. Upregulation of TrKA can be caused either by NTRK1 gene fusion or intact TrKA protein overexpression causing oncogenesis in multiple tumors including NSCLC. VMD-928 is the first oral small-molecule TrkA (NTRK1) selective inhibitor. In the first in-human phase 1 trial (NCT03556228) 20 patients were accrued to 4 dose escalation cohorts ranging from 300 mg/day to 2400 mg/day. Common adverse events related to therapy were dark stool (35%), elevated liver enzymes (25%, primarily at 2400 mg/day), fatigue, nausea or vomiting, and decreased appetite (20% each). Tumor types with reported high TrkA protein expression including squamous cell carcinoma of the lung (71%) are being accrued [89]. Table 9 summarizes the ongoing phase I/II clinical trials involving NTRK inhibitors in patients with advanced NSCLC.

**Table 9:** Current ongoing early phase I/II trials with NTRK fusion inhibitors

NTRK gene fusion inhibitors				
Drug name	Mechanism of action	Clinical trial (phase)	Study design	Disease
Larotrectinib	TRK fusion inhibitor	NCT02576431 (2)	Monotherapy	Solid tumors including NSCLC with NTRK fusions
Repotrectinib	TRK, ROS 1, ALK fusion inhibitor	NCT03093116 (1)	Monotherapy	Solid tumor including NSLC with ALK, ROS1, NTRK1/2/3 gene rearrangement
Entrectinib	ROS1 and TRK fusion inhibitor	NCT02568267 (2)	Monotherapy	Solid tumors including NSCLC with NTRK fusions and ROS1 rearrangements
VMD-928	small-molecule TrkA (NTRK1) inhibitor	NCT03556228 (1)	Monotherapy	Advanced solid tumors and lymphoma including NSCLC

#### KRAS inhibitors

KRAS (Kirsten RAT Sarcoma Viral Oncogene Homologue) is the most frequently mutated oncogene in human cancers and encodes guanosine triphosphatase and regulates signal transduction [90]. KRAS mutation is often associated with resistance to targeted therapies and poor outcomes in patients [91]. The KRAS p.G12C mutation occurs in about 13% of NSCLC [92].

Sotorasib (AMG510) is a small molecule that inhibits KARS G12C through interaction with the P2 pocket. In a single-group, phase 2 trial, sotorasib was administered orally at a dose of 960 mg once daily, in patients with KRAS p.G12C–mutated advanced NSCLC previously treated with standard therapies. Among the 126 enrolled patients, the majority (81.0%) had previously received both platinum-based chemotherapy and inhibitors of programmed death 1 (PD-1) or programmed death ligand 1 (PD-L1). An objective response was observed in 46 patients (37.1%) including 4 (3.2%) who had a complete response. The median DOR was 11.1 months with DCR of 80.6%. The median PFS was 6.8 months and the median OS was 12.5 months. TRAEs occurred in 88 of 126 patients (69.8%), including grade 3 events in 25 patients (19.8%) and a grade 4 event in 1 (0.8%). The most frequent TRAEs were diarrhea (31.7%), nausea (19.0%), increase in the alanine aminotransferase level (15.1%), increase in the aspartate aminotransferase level (15.1%), and fatigue (11.1%) [93]. In the exploratory analyses of the phase 2 CodeBreak 100 trial, the clinical benefit of sotorasib was observed across patient subgroups including patients with STK11 and KEAP1 mutations (94). Sotorasib was approved by FDA for KRASG12C mutated NSCLC in May 2021 [95]. A global phase III randomized study comparing sotorasib to docetaxel in KRAS G12C mutant NSCLC (CodeBreak 200) has begun recruiting (NCT04303780).

Adagrasib (MRTX-849) is a small-molecule inhibitor of KRAS G12C that binds to cysteine 12 in the inducible switch II pocket of KRAS G12C, inactivating it and preventing oncogenic signaling. It is currently being evaluated in a phase I/II study [96]. In the updated results of Krystal-1 trial (NCT03785249), 79 patients with pretreated NSCLC were treated with Adagrasib 600 mg bid. The only grade 3/4 treatment-related serious adverse event was hyponatremia (3%, 2/79). Among the 51 patients evaluable for clinical activity (14 from Phase 1/1b; 37 from Phase 2), 45% of patients had an objective response. The disease control rate was 96% (49/51) [97]. In KRYSTAL-12 (NCT04685135) adagrasib versus docetaxel are being studied in a phase III trial in patients with previously treated NSCLC with KRAS G12C mutation. The study is designed to demonstrate improvement in the dual primary endpoints of progression-free survival (PFS) and overall survival (OS) [98].

Other KRAS inhibitors that are currently being developed and studied in phase I/II trials include GDC-6036 (NCT04449874), D-1553 (NCT04585035), and Rigosertib (NCT04263090). Table 10 summarizes the ongoing phase I/II clinical trials involving KRAS inhibitors in patients with advanced NSCLC.

**Table 10:** Current ongoing early phase I/II trials with KRAS inhibitors

KRAS inhibitors				
Drug name	Mechanism of action	Clinical trial (phase)	Study design	Disease
Adagrasib (MRTX849)	KRAS G12C Inhibitor	NCT04330664 (1/2)	Combination with TNO155	KRAS p.G12C mutant solid tumors including NSCLC
Adagrasib (MRTX849)	Irreversible KRAS G12C Inhibitor	NCT04613596 (1/2)	Combination with Pembrolizumab	KRAS p.G12C mutant NSCLC with known PDL1 TPS score
GDC-6036	KRAS G12C Inhibitor	NCT04449874 (1)	Monotherapy	KRAS p.G12C mutant NSCLC
D-1553	KRAS G12C Inhibitor	NCT04585035 (1/2)	Monotherapy and combination with other standard therapies	KRAS p.G12C mutant NSCLC
Rigosertib	RAS-mimetic	NCT04263090 (1/2)	Combination with Nivolumab	KRAS mutant NSCLC with progression on fist line therapy
Sotorasib (AMG 510)	KRAS G12C Inhibitor	NCT03600883 (1/2)	Monotherapy	KRAS p.G12C mutant NSCLC
Sotorasib (AMG 510)	KRAS G12C Inhibitor	NCT04303780 (3)	Monotherapy	KRAS p.G12C mutant NSCLC

#### Drug antibody conjugates

Antibody–drug conjugates (ADCs) are cancer agents with a cytotoxic payload linked to a monoclonal antibody (mAB) that targets cancer cells.

Patritumab deruxtecan (HER3-DXd/U3-1402) is a HER3-targeted ADC with a fully humanized antibody, novel cleavable peptide-based linker, and topoisomerase I inhibitor payload via a tetrapeptide-based cleavable linker. It has demonstrated tolerable safety and antitumor activity in an ongoing study (NCT03260491) [99]. Efficacy and safety of patritumab deruxtecan in heavily pretreated 57 metastatic NSCLC patients with progressive disease on EGFR TKI was presented in ASCO 2021. At a dose of 5.6 mg/kg IV Q3W with median follow-up of 10.2 months confirmed ORR was seen in 39% (22/57) with DCR of 72%. Median DOR was 6.9 months, and median PFS was 8.2 mo. Antitumor activity was observed across diverse mechanisms of EGFR TKI resistance, including those not directly related to HER3 (EGFR C797S, MET or HER2 amp, and BRAF fusion). The most common grade ≥ 3 adverse events (AEs) were thrombocytopenia (30%), neutropenia (19%), and fatigue (14%). Drug-related interstitial lung disease occurred in 4 pts. 6/57 pts (11%) had AEs associated with treatment discontinuation [100].

Cofetuzumab pelidotin (ABBV-647) is an anti-PTK7 antibody–drug conjugate comprising the hu6MO24 monoclonal antibody, a cleavable cysteine-reactive linker, and Aur0101 (an auristatin microtubule inhibitor) [101]. Protein tyrosine kinase 7 (PTK7) is a highly conserved receptor tyrosine kinase involved in the Wnt signaling pathway and is overexpressed in multiple cancer types, including NSCLC [102]. A phase 1 trial previously showed promising antitumor activity and manageable safety in advance solid cancer including NSCLC. DCR of 56% was seen in 25 heavily pretreated NSCLC patients [103]. A phase 1b study (NCT04189614) is currently enrolling 40 recurrent NSCLC patients expressing PTK7 to study its safety and tolerability [101].

Carcinoembryonic antigen-related cell adhesion molecule 5 (CEACAM5) is a cell-surface glycoprotein highly expressed in NSCLC. SAR408701 is a DM4 conjugated ADC targeting CEACAM5. Updated safety and efficacy data for SAR408701 from the expansion part of the first-in-human study was reported in NSCLC patients. Two cohorts of patients including moderate (CEACAM5 expression at ≥ 2 + intensity between ≥ 1% to < 50%) and high expression (≥ 50% of the tumor cell population) were analyzed. ORR was 7.1% in the moderate expression group and 20.3% in the high expression cohort. Grade ≥ 3 TEAEs were found in 47.8% of pts [104]. A phase 3 trial is underway (NCT04154956).

TROP2 is an intracellular calcium signaling transducer overexpressed in NSCLC, portending poor survival. DS-1062 (datopotamab deruxtecan) is an ADC consisting of a humanized anti-TROP2 IgG1 monoclonal antibody attached to a topoisomerase I inhibitor payload via a tetrapeptide-based cleavable linker [105]. Results from the ongoing phase 1 study (TROPION-PanTumor01; Spira, WCLC 2020) demonstrated an overall response rate (ORR) of 21%, a disease control rate (DCR) of 67%, and a preliminary median progression-free survival (PFS) of 8.2 months (all by BICR), with a manageable safety profile, in patients with NSCLC who were treated with 6 mg/kg of Dato-DXd. The most common TEAEs were reported in 128 patients (96%); most frequent TEAEs (≥ 30%) included nausea (50%), stomatitis (44%), alopecia (40%), and fatigue (33%). Sixty-four patients (48%) experienced grade ≥ 3 TEAEs (most frequently dyspnea [5%]). There were 12 patients (9%) with interstitial lung disease [105]. A randomized, phase 3 study of DS-1062 versus docetaxel in previously treated advanced or metastatic non-small cell lung cancer (NSCLC) without actionable genomic alterations (TROPION-Lung01) is currently also underway (NCT04656652) [106].

MGC018 is an investigational ADC with a Duocarmycin payload linked to an anti-B7-H3 mAB. It is hypothesized that MGC018 has activity against B7-H3 expressing tumors (NCT03729596) [107]. Table 11 summarizes the ongoing phase I/II clinical trials involving ADC in patients with advanced NSCLC.

**Table 11:** Current ongoing early phase I/II trials with ADC

ADC				
Drug name	Mechanism of action	Clinical trial (phase)	Study design	Disease
CAB-ROR2-ADC	Anti-ROR2 ADC	NCT03504488 (1/2)	Monotherapy	Advanced solid tumors including NSCLC
Cofetuzumab Pelidotin	PTK7 ADC	NCT04189614 (1b)	Monotherapy	PTK7-expressing recurrent NSCLC
DS-1062a	ADC targeting TROP2	NCT04612751 (1)	Combination with Durvalumab	Advanced NSCLC with progression on chemotherapy and immunotherapy
MGC018	ADC delivers Duocarmycin	NCT03729596 (1/2)	Monotherapy and combination with MGA012 (anti-PD1	Advanced solid tumors including NSCLC
U3-1402 (Patritumab deruxtecan)	Drug (MAAA-1181a) linked to AB (Patritumab)	NCT03260491 (1)	Monotherapy	Advanced NSCLC with acquired resistance to EGFR TKI

#### PI3K/AKT/mTORC inhibitors

The PI3K/AKT/mTOR pathway has been heavily implicated in both tumorigenesis and progression of disease in NSCLC. In a study, 1144 patients with NSCLC underwent next generation sequencing for PI3KCA mutations. Mutations were identified in 3.7% of the patients [108]. Non-small cell lung squamous cell carcinoma (8.9%) were more likely to be have these mutations when compared with adenocarcinoma (2.9%). On the other hand upregulation of mTOR pathway is seen in up to 90% of the patients with NSCLC adenocarcinoma compared to 40% of the patients with squamous cell carcinoma [109]. S6K and 4E-BP1 are the downstream products of mTOR activation. These have also been identified in up to 58% and 25% of NSCLC specimens respectively [110]. It is also postulated that in patients with an EGFR mutant NSCLC, the AKT/mTOR pathway is constitutively activated in 67% of cases [111].

Sapanisertib (TAK228) is an oral TORC1/2 inhibitor. In a phase II trial with stage IV squamous NSCLC patients, it showed differential activity in NFE2L2 (ORR of 20%) and KEAP1(ORR of 17%) mutant patients [112]. Most common AEs included hyperglycemia (72%), fatigue (32%), diarrhea (32%), decreased appetite (32%).

Sirolimus binds to FKBP-12, an intracellular protein, to form an immunosuppressive complex which inhibits mTOR [113]. It is being studied in a phase I/II trial NCT01737502 with auranofin (gold salt) in advanced or recurrent NSCLC patients without standard treatment options. Primary outcomes include maximal tolerated dose of auranofin, number and severity of adverse events and progression free survival.

Gedatolisib (PF-05212384) is an intravenous, ATP-competitive, pan-PI3K/mTORi that has demonstrated significant antitumor activity in preclinical models. A first in-human study (NCT00940498) evaluated the safety, tolerability, PK, PD, and preliminary activity of gedatolisib in 78 patients with advanced solid tumors [114]. This study demonstrated manageable safety, with stomatitis and nausea as the most frequent AEs. There were two PRs in this heavily pretreated patient population. A phase I clinical trial (NCT03065062) of PI3K/mTOR inhibitor Gedatolisib in combination with CDK4/6 inhibitor Palbociclib is currently recruiting patients with advanced solid cancer patients including squamous NSCLC.

RMC-5552 is a bi-steric mTORC1-selective inhibitor [115]. It is currently being evaluated in a phase I/Ib, open label, multicenter, dose escalation trial (NCT04774952) as monotherapy in adults with relapsed refractory solid tumor including NSCLC.

Nab-Rapamycin (ABI-009), an mTOR inhibitor, is under investigation in a phase I/II trial with Nivolumab in patients with advanced solid tumors, including NSCLC (NCT03190174).

In combination with Pembrolizumab, Idelalisib, a PI3K inhibitor, is being looked at in patients with advanced NSCLC with progression on first-line therapy (NCT03257722). Table 12 summarizes the ongoing phase I/II clinical trials involving mTOR/P13K inhibitors in patients with advanced NSCLC.

**Table 12:** Current ongoing early phase I/II trials with mTOR/PI3K inhibitors

mTOR/PI3K pathway Inhibitors				
Drug name	Mechanism of action	Clinical trial (Phase)	Study design	Disease
Gedatolisib (PF-05212384)	pan-PI3K/mTOR inhibitor	NCT03065062 (1)	Combination with Palbociclib	Advanced tumors including squamous NSCLC
Idelalisib	PI3K inhibitor	NCT03257722 (1)	Combination with Pembrolizumab	Advanced NSCLC with progression on first line therapy
Nab Rapamycin (ABI-009)	mTOR inhibitor	NCT03190174 (1/2)	Combination with Nivolumab	Advanced solid tumors including NSCLC
RMC-5552	bi-steric mTORC1-selective inhibitor	NCT04774952 (1/1b)	Monotherapy	Advanced solid tumors including NSCLC
Sapanisertib	mTORC1/mTORC2 inhibitor	NCT02417701 (2)	Monotherapy	KRAS mutant NSCLC with KEAP1 mutation
Sirolimus	mTOR inhibitor	NCT01737502 (1/2)	Combination with Auranofin	Advanced or Recurrent Lung Cancer

#### Miscellaneous target inhibitors

##### ERBB2 inhibitors

Human epidermal growth factor receptor 2 (HER2, ERBB2) activating mutations occur in 2% of lung cancers (116). Ado-trastuzumab emtansine (T-DM1) is a HER2 targeted antibody–drug conjugate. NCCN recommends T-DM1 as an option for patients with ERBB2 mutated NSCLC patients based on a phase II basket trial [117].

Trastuzumab deruxtecan (T-DXd; DS-8201) is currently being evaluated in DESTINY-Lung01 trial (NCT03505710). Interim results presented in ASCO 2020 showed an ORR of 61.9% (95% CI 45.6–76.4%) with a DCR of 90.5% (95% CI 77.4–97.3%) in pretreated population; estimated median PFS was 14.0 month (95% CI 6.4–14.0 month). TRAE caused drug discontinuation in 10 patients (23.8%). HER2 mutations were predominantly in the kinase domain (90.5%) [118].

Combination of trastuzumab, pertuzumab and docetaxel was studied in as phase II IFCT-1703 R2D2 trial which enrolled patients with advanced NSCLC harboring HER2 mutation after progression of disease on ≥ 1 platinum based chemotherapy. In the 44 evaluable patients, ORR was 29% (*n* = 13) and stable disease was seen in 56% (*n* = 26) of the patients. Median PFS was 6.8 months. Median duration of treatment in patients with confirmed response (*n* = 13) was 10 months. Grade 3/4 treatment-related adverse events (AEs) were observed in 64% of patients. Most frequent grade ≥ 3 AEs were neutropenia (33%), diarrhea (13%) and anemia (9%). Grade 1/2 dyspnea was observed in 3 (6.7%) patients [119].

##### SHP2 inhibitors

SHP2 is a non-receptor protein tyrosine phosphatase encoded by the PTPN11 gene and is involved in cell growth differentiation via the MAPK signaling pathway [120].

TNO155 is a highly potent first-in-class SHP2 inhibitor [121]. A phase 1 dose-finding study of TNO155, with advanced solid tumors including EGFR or KRAS G12 mutant NSCLC, is currently recruiting patients (NCT03114319).

JAB-3068 is an oral SHP2 inhibitor being studied in a first-in-human study in patients with advanced solid tumors including NSCLC, in a phase I/II clinical trial (NCT03565003).

##### Others

Aliseritib is a selective aurora A kinase inhibitor currently under investigation in a phase I/II trial NCT04085315 with osimertinib in patients with EGFR mutant NSCLC progressing after first-line therapy. APG-1252 is a small molecule BCL2 inhibitor undergoing phase 1 trial NCT04001777 as monotherapy or in combination with osimertinib. APR0246 (Eprenetapopt) stabilizes p53 in normal functional structure and is being evaluated in advanced solid tumors, including NSCLC combined with Pembrolizumab in a phase I/II trial NCT04383938. A first inhuman phase 1 study of novel PARP7 inhibitor RBN-2397 in patients with advanced solid cancers including NSCLC showed tolerability, dose dependent increases in plasma exposures, evidence of target inhibition, and preliminary signs of clinical activity [122]. Study expansion is planned to evaluate safety and efficacy in squamous NSCLC, HNSCC, HR + breast cancer, and PARP7 amplified tumors (NCT04053673). Table 13 summarizes the ongoing phase I/II clinical trials involving miscellaneous target inhibitors in patients with advanced NSCLC.

**Table 13:** Current ongoing early phase I/II trials with miscellaneous target inhibitors

Drug name	Mechanism of action	Clinical trial (phase)	Study design	Disease
*ERBB2(HER2) inhibitors*				
Ado-trastuzumab emtansine	HER2-targeted antibody–drug conjugate	NCT03784599 (1/2)	Combination with osimertinib	EGFR mutant advanced NSCLC
Mobocertinib (TAk-788)	TKI targeting EGFR ex20ins mutations	NCT02716116 (1/2)	Combination with chemotherapy	EGFR and HER2 exon 20 insertion in advanced NSCLC
Poziotinib	EGFR, HER2 and HER 4 inhibitors	NCT03066206 (2)	Monotherapy	EGFR exon 20 and HER2 exon 20 mutated advanced NSCLC
trastuzumab deruxtecan	HER2-directed antibody–drug conjugate	NCT04644237 (2)	Monotherapy	HER2 mutated advanced NSCLC
*SHP2 inhibitors*				
JAB-3068	SHP2 inhibitor	NCT03518554 (1)	Monotherapy	Advanced solid tumors including NSCLC
TNO155	SHP2 inhibitor	NCT03114319 (1)	Monotherapy and combination with Nazartinib	Advanced solid tumors including NSCLC
*Other miscellaneous target inhibitors*				
Aliseritib	Selective aurora A kinase inhibitor	NCT04085315 (1/2)	Combination with Osimertinib	EGFR mutant NSCLC progressing after first line TKI
Anlotinib	VEGFR, FGFR, PDGFR, c-kit Inhibitor	NCT04165330 (1/2)	Combination with Nivolumab	Solid tumors including NSCLC
Apatinib	VEGFR-2 Inhibitor	NCT03811054 (2)	Combination with EGFR-TKI	EGFR mutant NSCLC progressing after first line TKI
APG-1252	Small molecule inhibitor of BCL2	NCT04001777 (1)	Monotherapy and combination with Osimertinib	EGFR mutant NSCLC
BMS-986205	IDO1 inhibitor	NCT02658890 (1/2)	Combination with Nivolumab and Ipilimumab	Advanced solid tumors including NSCLC
Eprenetapopt (Apr-246)	Stabilizes p53 in normal, functional structure	NCT04383938 (1/2)	Combination with Pembrolizumab	Advanced solid tumors including NSCLC
HBI-8000	Histone deacetylase inhibitor	NCT02718066 (1/2)	Combination with Nivolumab	Advanced NSCLC
Idelalisib	PI3K inhibitor	NCT03257722 (1/2)	Combination with Pembrolizumab	Advanced NSCLC with progression on first line therapy
IRX4204	Rexinoid, potent activator of RXRs	NCT02991651 (1)	Monotherapy and combination with Erlotinib	Advanced NSCLC with progression on two lines of therapy
NC318	IgG1 mAB specific for S15	NCT03665285 (1/2)	Monotherapy	Advanced solid tumors including NSCLC
Ningetinib (CT053PTSA)	Multi kinase inhibitor MET/HGFR	NCT03758287 (1/2)	Combination with Gefitinib	NSCLC with EGFR mutation and T790M negative
Nintedanib	PDGFR, FGFR, VEGFR inhibitor	NCT03377023 (1/2)	Combination with Nivolumab and Ipilimumab	Advanced or metastatic NSCLC
RGX-104	Liver X receptor agonist	NCT02922764 (1)	Monotherapy or in combination with immunotherapy or chemotherapy	Advanced solid tumors including NSCLC
Rucaparib	PARP inhibitor	NCT03845296 (2)	Monotherapy	Recurrent NSCLC
Selinexor	Exportin 1 inhibitor	NCT03095612 (1/2)	Combination with docetaxel	Advanced KRAS mutant NSCLC with progression on first line therapy
Selumetinib	MAPK inhibitor	NCT03392246 (2)	Combination with Osimertinib	Advanced EGFR mutant NSCLC
Ramucirumab	VEGFR2 antagonist	NCT03909334 (2)	Combination with Osimertinib	EGFR mutant NSCLC
Telaglenastat HCL	Glutaminase inhibitor	NCT03831932 (1/2)	Combination with Osimertinib	Advanced EGFR mutant NSCLC
Varlilumab	Anti CD27 antibody	NCT04081688 (1)	Combination with Atezolizumab	Advanced NSCLC with progression on first line therapy
Vorolanib	VEGFR/PDGFR inhibitor	NCT03583086 (1/2)	Combination with Nivolumab	Solid tumors including NSCLC
ZN-c3	WEE1 inhibitor	NCT04158336 (1/2)	Combination with Talazoparib or Pembrolizumab	Advanced solid tumors including NSCLC
RBN-2397	PARP7 inhibitor	NCT04053673 (1)	Monotherapy	Advanced solid tumors including NSCLC

## Conclusion

Lung cancer mortality has improved in the last decade largely due to the development of novel agents targeting actionable mutations in cancer cells. Although these drugs have revolutionized our clinical practice, resistance to these agents remains a reality. Identifying escape pathways and utilizing next-generation drugs by themselves or in combination can help us overcome this problem.

## Data Availability

The material supporting the conclusion of this review has been included within the article.

## Abbreviations

NSCLC: Non-small cell lung cancer
EGFR: Endothelial growth factor receptor
ALK: Anaplastic lymphoma kinase
PFS: Progression free survival
ORR: Overall response rate
OS: Overall survival
TRAE: Treatment related adverse effects
TEAE: Treatment emergent adverse effects
